# Unraveling Executive Functioning in Dual Diagnosis

**DOI:** 10.3389/fpsyg.2016.00979

**Published:** 2016-06-28

**Authors:** Judith C. L. M. Duijkers, Constance Th. W. M. Vissers, Jos I. M. Egger

**Affiliations:** ^1^Centre of Excellence for Korsakoff and Alcohol Related Cognitive Dysfunctions/Addiction Care, Vincent van Gogh Institute for PsychiatryVenray, Netherlands; ^2^Behavioural Science Institute, Radboud University NijmegenNijmegen, Netherlands; ^3^Kentalis Academy, Royal Dutch KentalisSint-Michielsgestel, Netherlands; ^4^Centre of Excellence for Neuropsychiatry, Vincent van Gogh Institute for PsychiatryVenray, Netherlands; ^5^Donders Institute for Brain, Cognition and Behaviour, Radboud University NijmegenNijmegen, Netherlands; ^6^Pompe Institute for Forensic Psychiatry, Pro PersonaNijmegen, Netherlands

**Keywords:** executive functioning, dual-diagnosis, comorbidity, substance use disorder, alcohol use disorder, addiction, schizophrenia, bipolar

## Abstract

In mental health, the term dual-diagnosis is used for the co-occurrence of Substance Use Disorder (SUD) with another mental disorder. These co-occurring disorders can have a shared cause, and can cause/intensify each other’s expression. Forming a threat to health and society, dual-diagnosis is associated with relapses in addiction-related behavior and a destructive lifestyle. This is due to a persistent failure to control impulses and the maintaining of inadequate self-regulatory behavior in daily life. Thus, several aspects of executive functioning like inhibitory, shifting and updating processes seem impaired in dual-diagnosis. Executive (dys-)function is currently even seen as a shared underlying key component of most mental disorders. However, the number of studies on diverse aspects of executive functioning in dual-diagnosis is limited. In the present review, a systematic overview of various aspects of executive functioning in dual-diagnosis is presented, striving for a prototypical profile of patients with dual-diagnosis. Looking at empirical results, inhibitory and shifting processes appear to be impaired for SUD combined with schizophrenia, bipolar disorder or cluster B personality disorders. Studies involving updating process tasks for dual-diagnosis were limited. More research that zooms in to the full diversity of these executive functions is needed in order to strengthen these findings. Detailed insight in the profile of strengths and weaknesses that underlies one’s behavior and is related to diagnostic classifications, can lead to tailor-made assessment and indications for treatment, pointing out which aspects need attention and/or training in one’s self-regulative abilities.

## Introduction

In dual-diagnosis, a Substance Use Disorder (SUD) co-occurs with another psychiatric condition such as psychotic disorder, mood disorder, anxiety disorder or personality disorder (Ziedonis and Brady, 2005). The Epidemiologic Catchment Area study, a comprehensive study of comorbidity, showed that the lifetime SUDs-rate in the general population was 17%, compared to 48% for persons with schizophrenia and 56% for persons with bipolar disorder (Regier et al., 1990). SUD is described in respectively 27 and 24% of patients with a depressive disorder or an anxiety disorder (Mueser et al., 2011; Dom et al., 2013). Studying a dual-diagnosis population is relevant, because of the threat that SUD and dual-diagnosis form to health, society, and the presence of relapses in addiction-related behavior and destructive lifestyles (De Jong et al., 2006; World Health Organisation [WHO], 2011). In clinical practice dual-diagnosis frequently occurs whereas ideally distinguishable single disorder groups are rare. Disorders in dual-diagnosis can have a shared cause, or can cause/intensify each other’s expression (Mueser et al., 1998). It is not always easy or possible to distinguish in which way the disorders causally interact or influence each other.

In most SUDs and other mental disorders self-regulatory behavior to manage daily life situations (involving work and relationships) falls short. Coping strategies are impaired, resulting in affective breakdowns (American Psychiatric Association, 2013). These frequently observed symptoms of psychiatric disorders point directly to deficits in executive functioning (EF) (among others: Barkley, 2001; Egger et al., 2007; Fernandez-Serrano et al., 2010; Wiers et al., 2012; Janssen, 2013; Luijten et al., 2013; Thoma and Daum, 2013; Goschke, 2014; Smith et al., 2014; Snyder et al., 2015). Executive (dis)-functioning is currently even seen as a shared underlying key component of most mental disorders (Egger et al., 2007; Janssen, 2013; Goschke, 2014; Snyder et al., 2015).

Executive functioning can be defined as all cognitive processes that regulate behavior in such a manner that it can be efficient and goal-orientated (Miyake et al., 2000; Barkley, 2001; Friedman et al., 2008; Miyake and Friedman, 2012; Snyder et al., 2015). Barkley (2001) describes EF as serving to “shift the control of behavior from the immediate context, social others, and the temporal now to self-regulation by internal representations regarding the hypothetical social future”. Miyake et al. (2000) introduced a model of EF in which three key EF aspects were presented. Firstly, *Shifting* concerns the switch of attention between tasks/operations and/or mental sets. Translated into daily life, it involves mental flexibility to repeatedly let go of irrelevant and/or inappropriate behaviors (for example, drug use or attention bias to alcohol related cues) and switch to more adequate/relevant behaviors (like sporting or switch of television-channel). Secondly, *Updating* involves the process of actively manipulating and monitoring relevant information in working memory, in order to keep track of information that is old and needs actualization (Miyake et al., 2000). For SUD, it can involve craving. When longing for drugs, a patient may usually tend to call the drug dealer. But, in a recent relapse prevention session he or she learned about putting the numbers of supporting friends in their phone. As this is the first moment of intense craving after the session and intrusive substance-use thoughts already come to mind, the patient has to monitor his or her own behavior and promptly update these thoughts by thinking of the newly learned information and healthier thoughts. He or she needs to replace the old information about the drug dealer with the new information regarding their supportive friend’s phone number. *Inhibition* is also needed in this scenario, and it is defined as the ability to inhibit dominant, automatic responses when necessary (Miyake et al., 2000; Barkley, 2001). *Inhibition is*, for instance, the suppression of approaching alcohol/drugs and/or calling the drug dealer. *Shifting, updating* and *inhibition* are all needed to some extent when making daily life decisions. For instance, when one wants to succeed in arriving at work on time tomorrow, thereby stopping destructive-avoidant behavior that was linked to one’s SUD lifestyle: (i) *shifting* is needed to get up earlier than before and to repeatedly let go of attention biases triggering late night out fantasies, (ii) *inhibition* is needed to prevent this late night ‘going out’ with friends by stopping yourself from drinking/using and going home, (iii) *updating* is involved in checking an alternative work-route, thereby avoiding and replacing the old, coffee-shop route that would trigger craving. The interplay between EF aspects can influence self-regulation in daily life by reducing problem-behavior and raising more goal-directed behavior. In addition, EF can facilitate one’s controlled coping with negative feelings and externalizing problem behaviors like substance-abuse/aggressive outbursts. On the contrary part, executive *dys*function can cause/aggravate negative feelings/behavioral outbursts by the perceived lack of control (Goschke, 2014).

**FIGURE 1:**
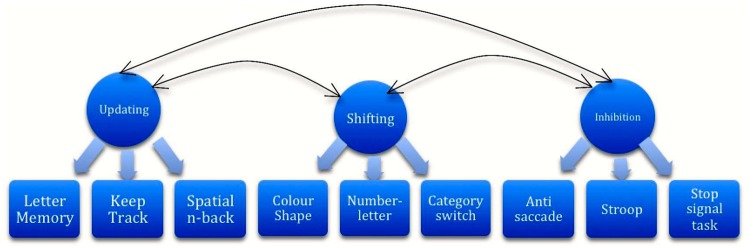
**Unified and diverse aspects of executive functioning (Snyder et al., 2015.**)

The both unified and diverse aspects of EF can be placed in a model (Snyder et al., 2015, p. 13). EF can be assessed by global or more specific neurocognitive tasks (**Figure 1** for specific tasks). If severe executive dysfunctioning is expected, the use of both global and specific EF tasks is recommended by Snyder et al. (2015). *Inhibition* can be measured by Go–NoGo, Stop Signal, Approach Avoidance Tasks, the Stroop, Anti-saccade, Event Related Potential components like P300, self-report/rating measures such as the Frontal Systems Behavioral Scale, BIS-BAS scales (Behavioral Inhibition System-Behavioral Approach System) and by the MMPI-2 Impulse-Control index^1^ (Stroop, 1935; Hallett, 1978; Butcher et al., 1989; Carver and White, 1994; Robbins et al., 1994; Roberts et al., 1994; Rogers and Monsell, 1995; Mayr and Kliegl, 2000; Grace and Malloy, 2001; Miyake et al., 2004; Handy, 2005; Franken et al., 2006; Friedman et al., 2008; Wiers et al., 2009; Wiers et al., 2010). *Shifting* can be measured by the Trail Making Test (Reitan, 1958), the Wisconsin Card Sorting Test (Berg, 1948), and other Category Switching Tasks. *Updating* can be measured by the letter memory task (Morris and Jones, 1990), keep track task (Yntema, 1963) or spatial n-back task.^1^ *Updating* tasks involve monitoring of incoming information to check task-relevance, and replacement, update, of old non-relevant information by new information^1^ (Snyder et al., 2015).

Impairments in EF aspects can trigger the appearance of mental disorders by several mechanisms such as (i) a failure to maintain goals when confronted with interfering desires that are difficult to *inhibit* and/or complicate *shifting* to more healthy goals, (ii) *inhibitory* impairment of impulsive responses, (iii) sticking attention to disorder-linked cues like substances that interfere with adequate *shifting*, (iv) impaired cognitive control (*inhibition*) and distorted anticipational planning, (v) reduced (emotional) stress regulation, and (vi) cognitive *in*flexibility (Goschke, 2014). These mechanisms negatively interfere with adequate impulse control and decision making, consequently also thwarting proper self-regulatory behavior in daily life.

Executive functioning impairments were described in several mental conditions like schizophrenia, bipolar-, anxiety-, personality-, developmental disorder, and SUD. For the separate disorders, a high number of studies have been undertaken and multiple reviews are present (see, among others, Verdejo-Garcia et al., 2006; Egger et al., 2007; Fernandez-Serrano et al., 2010; Wiers et al., 2012; Luijten et al., 2013; Thoma and Daum, 2013; Goschke, 2014; Smith et al., 2014; Snyder et al., 2015). Elaborating on this, one can expect that in dual-diagnosis EF will also be impaired. The majority of SUD-patients and half of schizophrenia/bipolar disorder patients are dually diagnosed. That makes insight into EF profiles for dual-diagnosis highly relevant, striving to unravel strengths and pitfalls for daily life behavior. For instance, the indications for treatment differ if one’s pitfall primarily is the *inhibition* of undesired responses, or a flexible *shift* from one behavioral strategy to another. Recommendations for treatment can be formed when one oversees the differentiated profile of strengths and weaknesses, particularly in EF.

This article presents an overview of EF studies in dual-diagnosis.

## Materials and Methods

The inclusion criteria for studies were as follows: (i) A Method section that contains information concerning: (a) gender, age, test-materials and pre-assessment abstinence period of patients, and (b) diagnostic procedures that were used to determine if a dual-diagnosis was present or not; (ii) Comparisons by use of a healthy control group and/or a group without dual-diagnosis; and (iii) An abstinence period involving less risk for interfering influence of (sub-) acute substance effects, in order to only measure residual effects. The substance of study should not be traceable anymore. Studies with an abstinence period of at least one week were included. For cannabis, a period of four weeks was adhered to (for substance detection times see Verstraete, 2004; for interfering effects see Walvoort et al., 2013). Guidelines for PRISMA analysis were used to select papers (Moher et al., 2009). PRISMA analysis for Web of Science and PubMed resulted in 155 papers including the primary search terms of Executive Functioning AND dual-diagnosis. Four additional papers were found using other search term-combinations, and two additional papers were found by other sources such as on topic reviews (Rabin et al., 2011; Benaiges et al., 2010; Balanza-Martinez et al., 2015). Consequently, a total of 161 papers were screened (including duplicates). After screening, 131 papers were excluded for the following reasons: 121 papers involved the use of search terms in off-topic contexts: for instance, “dual” in “dual-task”. Five studies concerned theoretical/qualitative research/reviews and five papers involved medical/different use of the dual-diagnosis term. 30 articles were assessed for eligibility. Of these, 19 were excluded after analysis, for the following reasons: 12 papers did not mention abstinence periods, two papers’ method-sections did not contain gender/age/test materials, one paper was not found in full-text despite contacting authors and four papers were duplicates between searches. Finally, a total of 11 studies were selected based on the criteria (**Table 1**). Search terms were Executive Functioning, dual-diagnosis, Substance Use Disorder, alcohol use disorder, inhibition, updating, shifting, comorbidity, schizophrenia, bipolar disorder, personality disorder, anxiety disorder, mood disorder, developmental disorder, and addiction. The independent variable was the dual-diagnosis; the dependent variable was the level of functioning on EF tasks.

**Table 1:** Findings.

Dual-diagnosis	Authors	Tasks	*N*	Abstinence	Significant results
Schizophrenia and SUD	Benaiges et al., 2013	Trail Making Test (*Shifting*), Tower of Hanoi (*EF general*), Wisconsin Card Sorting Test (*Shifting, EF general*), Backwards digit subtest (*Working memory*), Iowa Gambling Task (*Decision Making*), Vocabulary (Other: premorbid verbal IQ)	30 DD 30 Schizophrenia without SUD 35 SUD	4 months	DD and SUD > Schizophrenia without SUD on: ^∗^*Shifting* (*P* < 0.05, Effect Size 0.08) ^∗^*Number of errors* (*P* < 0.01, Effect Size 0.13) ^∗^Speed (*P* < 0.01, Effect Size 0.10) SUD > Schizophrenia without SUD on: ^∗^*Decision making* (*P* < 0.05, Effect Size 0.07)

Schizophrenia and SUD	Jockers-Scherübl et al., 2007	Trail Making Test (*Shifting*), Wisconsin Card Sorting Test (*Shifting, EF general*), Continuous Performance Test (Other), Wechsler Memory Scale (Other), WAIS-R subtests (Other)	19 DD 20 Schizophrenia without SUD 21 HC 18 SUD	28 days	#Premorbid IQ difference and education level (DD < SUD) (IQ *F* = 7.88, *P* = 0.00) #No Effect Sizes reported DD = Schizophrenia without SUD on: all measures (*Shifting, EF General*, Other) DD = SUD on: ^∗^*Shifting* (Wisconsin Card Sorting Test) DD < SUD on: ^∗^*Shifting* (Trail Making Test) (*F* = 6.15, *P* = 0.02) ^∗^WAIS Comprehension (*F* = 18.71, *P* = 0.00) Picture arrangement (*F* = 4.45, *P* = 0.04) Digit Symbol (*F* = 4.66, *P* = 0.04) ^∗^Verbal Memory(*F* = 11.29, *P* = 0.00) ^∗^Attention (Symbols *F* = 5.49, *P* = 0.03; Digits *F* = 6.80, *P* = 0.01)

Schizophrenia and SUD	Rodriguez-Jiminez et al., 2010	Wisconsin Card Sorting Test (*Shifting, EF general*), Trail Making Test (*Shifting*), Stroop (*Inhibition*)	82 DD 121 Schizophrenia without SUD	1 month	#DD patients significantly younger age, more males and more psychotic episodes #No Effect Size reported #Age difference groups is factor of influence DD = Schizophrenia without SUD on: most executive measures (*Shifting, EF General*) DD < Schizophrenia without SUD on *Inhibition* task *P* = 0.015

Schizophrenia and SUD	Rodriguez-Jimenez et al., 2008	Wisconsin Card Sorting Test (*Shifting, EF general*)	65 DD 48 Schizophrenia without SUD	30 days	DD = Schizophrenia without SUD on: ^∗^*Shifting* (*F* = 0.382, *P* = 0.538) ^∗^*Perseverative Errors* (*F* = 0.396, *P* = 0.530)

Schizophrenia and SUD	Schnell et al., 2009	Trail Making test (*Shifting*), Verbal Fluency (*EF general*), Dual-Tasking (*EF*), Letter Number Span (*Working Memory*), Digit Symbol Test (Other), Auditory Verbal Learning and Memory Test (Other)	35 DD 34 Schizophrenia without SUD	3 weeks	DD > Schizophrenia without SUD on ^∗^*Executive Function* *^∗^Working Memory* (Sum tasks *T* = 2.923, *P* = 0.005) (Max. span *T* = 2.349, *P* = 0.022) ^∗^*Shifting* (*T* = -2.590, *P* = 0.012) ^∗^Verbal memory (Delayed recall *T* = 2.263, *P* = 0.027) (Recognition *T* = 2.246, *P* = 0.028) DD < Schizophrenia without SUD on academic achievement and vocabulary #Level of education in DD is lower than in Schizophrenia without SUD group #No Effect Sizes reported

Schizophrenia and SUD	Sevy et al., 2007	Trail Making Test (*Shifting*), Iowa Gambling Task (*Decision Making*), Controlled oral word association test (*EF*), CPT Identical Pairs test, Digit span subtest Wechsler Adult Intelligence Scale, California Verbal Learning test (all Other)	14 DD, 20 HC 13 Schizophrenia without SUD	1 week	#DD less education than Schizophrenia without SUD and HC group Schizophrenia (DD AND Schizophrenia without SUD) < HC on: ^∗^*Shifting* ability ^∗^Other measured neurocognitive functions (NB: Not study topic!) DD and Schizophrenia without SUD = HC on: ^∗^*Emotion-based decision-making* DD > Schizophrenia without SUD on: Digit span (*P* < 0.048)

Bipolar disorder and SUD	Marshall et al., 2012	Wisconsin Card Sorting Test (*Shifting, EF general*), Stroop (*Inhibition*), Parametric Go/No-Go task (*Inhibition*), FAS verbal fluency test (*EF*), Rey Complex Figure Test (*planning and other*), California verbal learning test-II, Purdue Pegboard test, Emotion Perception Test, Facial Emotion Perception Test, Digit Symbol Coding (all Other)	158 DD 97 HC 98 Bipolar without SUD	6 months	#Less education DD #No Effect Sizes reported DD < HC on: ^∗^*Conceptual Reasoning and set-Shifting* (*Shifting*) (*F* = 9.68, *P* = 0.001) ^∗^Processing Speed and interference resolution (*Inhibition*) (*F* = 15.88, *P* < 0.001) ^∗^*Inhibitory control* (*F* = 5.50, *P* = 0.007) ^∗^Visual Memory (*F* = 8.84, *P* = 0.001) ^∗^Fine Motor skill (*F* = 10.32, *P* < 0.001, DD < Bipolar without SUD on: ^∗^*Shifting* (*P* < 0.01) ^∗^Visual memory (*P* < 0.01) ^∗^Conceptual reasoning (*P* < 0.01)

Bipolar disorder and SUD	Van Gorp et al., 1998	Wisconsin Card Sorting Test (*Shifting, EF general*), Stroop (*Inhibition*), Trail Making Test (*Shifting*), FAS verbal fluency test (*EF*), Rey Complex Figure Test (*Planning and other*) California verbal learning test (Other), National Adult Reading Test (Other)	12 DD 22 HC 13 Bipolar without SUD	6 months	#No Effect Sizes reported DD < HC on: ^∗^*Shifting* (less finished categories on Wisconsin Card Sorting Test; *F* = 4.2469, *P* = 0.02) ^∗^*Errors* Wisconsin Card Sorting Test (*F* = 3.627, *P* = 0.04) ^∗^Verbal memory (*F* = 6.0427, *P* = 0.005) Bipolar without SUD < HC on: ^∗^Verbal memory (*F* = 6.0427, *P* = 0.005) ^∗^*Errors* Wisconsin Card Sorting Test (*F* = 3.627, *P* = 0.04)

Personality disorder and SUD	Albein-Urios et al., 2014	Letter-number sequencing (*Updating*), 2-back task (*Updating*), Delis-Kaplan Executive Function System Color Word Interference Test (*Inhibition and Shifting*), Category test (*Shifting*), D2 (Other)	37 DD 36 SUD 34 HC	>15 days	DD Cluster B PD < HC on: ^∗^*Attention/Inhibition* (*F* = 5.832, *P* < 0.001, Effect Size 1.0) (*F* = 2.866, *P* < 0.05, Effect Size 0.7) (*F* = 3.643, *P* < 0.05, Effect Size 0.7) ^∗^Concentration (*F* = 6.735, *P* < 0.001, Effect Size 1.1) DD Cluster C PD < HC on: ^∗^*Working memory* (*F* = 5.591, *P* < 0.001, Effect Size 1.1) ^∗^*Updating* (*F* = 4.021, *P* < 0.01, Effect Size 1.1) ^∗^Concentration (no significance digit Effect Size 1.0) ^∗^Efficiency (*F* = 5.433, *P* < 0.01, Effect Size 1.0) SUD < HC on: ^∗^*Shifting* (*F* = 3.818, *P* < 0.05, Effect Size 0.7) ^∗^*Working memory* (no significance digit Effect Size 1.0) ^∗^Concentration (no significance digit Effect Size 0.9) ^∗^Efficiency (no significance digit Effect Size 0.8)

Personality disorder and SUD	Albein-Urios et al., 2013	Letter-number sequencing (*Updating*), 2-back task (*Updating*), Stroop (*Inhibition*), Category test (*Shifting*), D2 (Other)	32 DD 44 SUD 34 HC	>15 days	DD < HC on: ^∗^*Shifting* (*F* = 6.59, *P* = 0.002, Effect Size 0.85) ^∗^*Updating* (*F* = 5.10, *P* = 0.008, Effect Size 0.76) ^∗^*Inhibition* (*F* = 5.98, *P* = 0.003, Effect Size 0.79) ^∗^Attention Total D2 score (*F* = 10.85, *P* = 0.000, Effect Size 1.01) Concentration D2 (*F* = 14.60, *P* = 0.000, Effect Size 1.24) Fluctuation D2 (*F* = 12.27, *P* = 0.000, Effect Size 1.05) DD < SUD on: ^∗^Attention (Effect Size 0.79, no significance digit) ^∗^*Inhibition* (Effect Size 0.50, no significance digit)

Conduct disorder and SUD	Giancola and Mezzich, 2000	Porteus Maze Test (*Planning)*, Vigilance Test (*Inhibition*), Motor restraint test (*Inhibition*), Stroop *(Inhibition)*, Test Language Competence (Other), Peabody Individual Achievement Test-Revised (Other), WISC/WAIS-R (Other)	239 DD 63 SUD (+other disorders) 58 Conduct dis. without SUD 110 HC	2 weeks	#SUD and Conduct Disorder groups also have other psychiatric disorders, so not “only” disorders DD < HC on: ^∗^*Inhibition* (*F* = 5.48, *P* < 0.01; *F* = 4.55, *P* < 0.01) ^∗^*Planning* (*F* = 4.41, *P* < 0.01) ^∗^Intelligence, ^∗^Language ^∗^Academic Achievement


*DD, Dual -diagnosis; HC, healthy controls; <functioning: worse level of functioning; >functioning: better functioning; SUD, Substance Use Disorder.*

## Results

Findings are presented for 11 studies that involve the dual-diagnoses of SUD-Schizophrenia (six), SUD-Bipolar disorder (two), SUD-Personality disorders (two) and SUD-Conduct disorder (one). For SUD-Anxiety and SUD-Developmental disorder no studies were found. Results are organized by disorder, tasks and EF aspect. Significance levels and effect sizes are described if present (**Table 1**).

### SUD-Schizophrenia

Rodriguez-Jiminez et al. (2010) performed the largest schizophrenia-SUD dual-diagnosis study, involving 82 patients with dual-diagnosis (Sch+) and 121 patients with Schizophrenia without SUD (Sch-). Results mostly showed comparable *Shifting* and EF general abilities for Sch+ and Sch-. The Sch+ group only functioned less at *Inhibitory* control as compared to the Sch- group. In two more studies, patients with Sch+ and patients with Sch- had similar results for several *shifting* tasks (Jockers-Scherübl et al., 2007; Rodriguez-Jimenez et al., 2008). Contradictory to those findings, two studies showed better functioning for patients with Sch+ as compared to Sch- on *shifting* abilities (Schnell et al., 2009; Benaiges et al., 2013). Furthermore, in one study, patients with Sch+ functioned less adequate at *shifting* (Trail Making Test) and *inhibition* tasks than patients with Sch- (Jockers-Scherübl et al., 2007). Lastly, Sevy et al., 2007; including a healthy control group) showed worse *shifting* abilities for Sch+ patients (schizophrenia-cannabis) as compared to healthy controls.

### SUD-Bipolar Mood Disorder

Patients with SUD-bipolar disorder showed poorer functioning on mental *shifting* ability as compared to healthy controls, measured by the Wisconsin Card Sorting Test. That is, dual-diagnosis patients completed fewer categories than controls (Van Gorp et al., 1998). Marshall et al. (2012) described that patients with SUD-bipolar disorder had more executive (*inhibitory control, set-shifting*, and *interference resolution*) dysfunctions than controls and patients with bipolar disorder without SUD.

### SUD-Personality Disorders

In 2013 and 2014, cocaine abusing patients with cluster B (borderline, narcissistic, histrionic, and anti-social) and cluster C (avoidant, dependent, and obsessive compulsive) personality disorders were studied at diverse EF. Patients with SUD-cluster B personality disorders, that is, more impulsive personality types, showed impairments in nearly all EF aspects as compared to controls and most specifically in *inhibitory control*. Patients with SUD-cluster C personality disorders, that is, more inhibited and obsessive personality types, showed more problems in *working memory* and *updating* ability. Patients with SUD as compared to the dual-diagnosis group had a better *inhibitory control*. However, compared to healthy controls, the SUD group also showed impairments in *shifting* and *working memory.* Patients with dual-diagnosis SUD-Personality disorders as a group consistently functioned less on *shifting, updating* and *inhibitory* abilities as compared to healthy controls (Albein-Urios et al., 2013; Albein-Urios et al., 2014).

### SUD-Conduct Disorder

One study compared 239 females with dual-diagnosis to healthy controls. Impairments were shown for the dual-diagnosis group on *inhibition* and *planning* ability as compared to healthy controls (Giancola and Mezzich, 2000).

### SUD-Anxiety Disorders, Developmental Disorders

Despite frequent co-occurrence of SUD-anxiety or SUD-developmental disorders (for prevalence numbers see Dom et al., 2013), we found no studies on EF for these groups.

## Discussion

### Aim and Model

Aim and Model Gaining a prototypical profile of EF for dual-diagnosis. This is expected to contribute to tailor-made directions for treatment.

### Findings

Findings Shifting and *inhibitory control* mostly are compromised in patients with dual-diagnosis as compared to healthy controls. If one were to think of dual-diagnosis and its overt behavioral symptoms, it is quite conceivable that dual-diagnosis implicates a high amount of (affective) turbulence and sensory sensitivity, since both substances and psychotic/bipolar/other symptoms have an interfering influence on the balance of several brain processes. Elaborating on this, maintaining a realistic view of daily life situations with flexible participation and properly timed inhibition when needed, is likely to be impaired. As compared to Sch-, patients with Sch+ show less *inhibitory control*. One possible explanation for this difference may be that patients with a SUD-combination show relatively more impulsivity, partly because the use of substances may negatively influence their dopaminergic *inhibitory* brain processes. Thereby, they seem to be less capable of inhibiting desires than patients without SUD. Furthermore, with a view to negative symptomatology, the schizophrenia patient-group is possibly more avoidant/*inhibited* than approaching in behavior manner. In terms of impulsivity, this hypothetical explanation also applies to the finding that patients with cluster B (impulsive type) personality disorder and SUD perform *inhibitory control* tasks less adequately than patients with only SUD. Whereas impulsive behavior has shown to negatively interfere with *inhibitory control*, it can also be influenced vice versa: common impulsive behavior in dual-diagnoses such as SUD-Bipolar disorder, SUD-cluster B Personality disorder and SUD-Conduct disorder (American Psychiatric Association, 2013) may be maintained or even urged by executive impairments. Impulsivity however seems to have a positive counterpart as well: the finding that Sch+ patients function slightly better on *shifting* abilities than Sch- patients may be linked to a higher tendency for impulsiveness and search for novelty in patients with SUD-combinations. This means that a flexible switch from familiar pathways to other routes may be easier for persons that are more impulsive and less rigid in behavior than for more avoidant persons that may seek and/or persevere in familiar styles of behavior.

### Limitations Concerning Dual-Diagnosis Research

Some conflicting findings reduce the certainty with which conclusions can be stated. These contradictions may be partly caused by factors that influenced several studies, and that may have restricted the validity and reliability of the observed empirical findings in those studies. For instance, the study of Jockers-Scherübl et al. (2007), that of Schnell et al. (2009) and that of Sevy et al. (2007) involved differences in education levels between the Sch+ (lower education level) and Sch- group. Furthermore, in one study the Sch+ group included younger patients and involved more males than the Sch- group. The differences in age actually showed to be a factor in differences found between the groups; so the observed weakened inhibitory control may be affected by the younger age of the dual-diagnosis group (study of Rodriguez-Jiminez et al., 2010). Complications in dual-diagnosis research are possibly due to several reasons. Sample sizes are mostly modest because it is difficult to recruit patients that (i) are in a mild psychiatric state needed for sufficient testability, (ii) are motivated to cooperate and stop using substances, and (iii) have achieved substance-abstinence for a reasonable time prior to assessment, ideally confirmed by tests. A pre-assessment abstinence period of at least six weeks is best, in case of alcohol, but probably for other substances as well, due to the recovery that the body undergoes in this period of time (Weeda et al., 2006; Walvoort et al., 2013). The length of substance abstinence and methods are not always clearly documented, which reduces the validity and reliability of conclusions. Furthermore, when testing patients with dual-diagnosis, it is not clear which disorder and/or substance contributes to which specific empirical finding (Balanza-Martinez et al., 2015). Partly for that reason, disorders and substances are usually studied “separately”. But procedures for dual-diagnosis presence are not always described. This makes it ambiguous whether separate disorders or dual-diagnosis is studied. For example, Giancola and Mezzich (2000) described a “SUD without dual-diagnosis” group in which no Conduct Disorder was present (2000); however, other psychiatric diagnoses were present in this “SUD” group, making it still a “dual-diagnosis” group.

### Future Research

Findings lead to the following future research recommendations. Firstly, studying dual-diagnosis has value, when methods are described in a valid manner. Secondly, sufficiently long abstinence periods before assessment, preferably approximately 6 weeks, should be attained to prevent findings from being influenced by acute or sub-acute substance use effects (Weeda et al., 2006; Walvoort et al., 2013). Thirdly, the use of healthy control groups is highly recommended to enable valid comparisons. Fourthly, studies need to be performed in patient groups that also exist in clinical practice, such as dual-diagnosis involving anxiety and developmental disorders. Fifthly, research on all diverse aspects of EF is recommended, also involving Updating processes/working memory. Impairments in flexible shifting abilities, updating processes, or impairments in inhibitory control over undesired responses will most probably lead to different indications for treatment. Finally, findings as described should get more strength through follow up research, including the test of the stated impulsivity hypothesis. Elaborating on these recommendations, there are promising results regarding EF treatment interventions, for instance with Dys-executive Syndrome Treatment Programs and Approach-Avoidance/Inhibitory Intervention Training (among others, Boelen et al., 2012; Rinck et al., 2013; Sharbanee et al., 2014). Hence, unraveling EF in dual-diagnosis has great value for coaching and treatment of patients, and it illustrates how the gap between neuroscience and psychotherapy can be bridged.

## Author Contributions

JD was first and corresponding author. She performed the literature search for this mini-review and wrote several concepts of the manuscript. She consulted CV on a regular basis as second author to revise concept manuscript versions and sharpen the described theoretical and empirical issues. CV revised concept versions and JE as last author was available for consultation during the project and revised the final concept version of the manuscript. In the end, the final manuscript and cover letter to be submitted was carefully revised by all three authors en JD eventually submitted the manuscript as first author.

## Conflict of Interest Statement

The authors declare that the research was conducted in the absence of any commercial or financial relationships that could be construed as a potential conflict of interest.

## Footnotes

1 Most of the tasks are described and categorized in a review by Snyder et al. (2015).
